# Hypothermic Effect of Acute Citral Treatment during LPS-induced Systemic Inflammation in Obese Mice: Reduction of Serum TNF-α and Leptin Levels

**DOI:** 10.3390/biom10101454

**Published:** 2020-10-17

**Authors:** Maycon T. Emílio-Silva, Vinicius P. Rodrigues, Gabriela Bueno, Rie Ohara, Marina G. Martins, José A. C. Horta-Júnior, Luiz G. S. Branco, Lúcia R. M. Rocha, Clélia A. Hiruma-Lima

**Affiliations:** 1 Department of Structural and Functional Biology (Physiology), Institute of Biosciences, São Paulo State University (UNESP), Botucatu, São Paulo 18618-970, Brazil; maycon.silva@unesp.br (M.T.E.-S.); viniciuspr42@gmail.com (V.P.R.); gabriela.sbueno17@gmail.com (G.B.); rieohara@gmail.com (R.O.); lucia.rocha@unesp.br (L.R.M.R.); 2 Department of Physiology, Institute of Biosciences, University of São Paulo (USP), São Paulo 05508-090, Brazil; marina.gmartins@gmail.com; 3 Department of Structural and Functional Biology (Anatomy), Institute of Biosciences, São Paulo State University (UNESP), Botucatu, São Paulo 18618-689, Brazil; anchieta.e@unesp.br; 4 Department of Basic and Oral Biology, Dental School of Ribeirão Preto, University of São Paulo (USP), Ribeirão Preto, São Paulo 14040-904, Brazil; branco@forp.usp.br

**Keywords:** obesity, lipopolysaccharide, systemic inflammation, monoterpene, citral

## Abstract

Citral is a mixture of monoterpenes present in the essential oil of several plants, such as *Cymbopogon citratus* and *Zingiber officinale*, possessing anti-inflammatory, anti-ulcerogenic, and antipyretic actions. We investigated the action of citral on body temperature (Tb) and inflammatory signaling in eutrophic and obese mice during Systemic Inflammation (SI) induced by Lipopolysaccharide (LPS). Thus, we assessed the effect of citral (25, 100, and 300 mg/kg) and ibuprofen in LPS-induced SI in Swiss male mice fed a standard diet (SD) or high-fat diet (HFD) for 12 weeks. Following SI induction, we measured Tb and collected the serum, hypothalamus, and gastric mucosa for biochemical measurements. Acute treatment with citral decreased the Tb of both SD and HFD-fed animals. Citral (300 mg/kg) treatment caused a significantly lower Tb variation in HFD-fed animals than in those fed the SD. Citral reduced peripheral levels of tumor necrosis factor (TNF)-α in SD and HFD mice and decreased serum leptin concentration in HFD mice 90 min after the LPS challenge. Furthermore, citral also reduced interleukin (IL)-6 levels in the hypothalamus of obese mice. In summary, citral effectively reduced Tb during SI by reducing inflammatory mediators with a distinct action profile in HFD mice when compared with SD.

## 1. Introduction

Obesity is a chronic disease that has been affecting different countries worldwide [1]. It is a complex and multifactorial disease that involves genetic, behavioral, socioeconomic, and environmental variables. The consumption of dietary fats is among the most important factors that lead to obesity. Ingestion of a high-fat diet (HFD) results in progressive body alterations and fat accumulation, triggering changes in the immune system characterized by an increase in systemic biomarkers such as interleukin (IL)-6, tumor necrosis factor (TNF)-α, and leptin [2,3,4].

The clarification that obesity involves a low-grade chronic inflammation has fundamentally changed the understanding of the underlying causes and progression of obesity. In obese patients, the low-grade systemic inflammatory status increases the levels of proinflammatory mediators, resulting in a greater susceptibility to infections caused by alterations in the host’s homeostasis [5,6].

Systemic inflammation (SI) occurs due to the body’s ability to promote protective responses against pathogenic infections through the identification of molecular patterns [6,7], as is the case of lipopolysaccharide (LPS), a constituent of the Gram-negative bacterial cell membrane. Its administration has been used as a well-accepted model to study SI [8], since it is capable of inducing inflammatory responses through activation of the innate immune system by interacting with Toll-like receptor (TLR)-4 [9]. This interaction triggers the production of peripheric inflammatory mediators, such as IL-1β, IL-6, and TNF-α, eventually signaling hypothalamic regions of the central nervous system (CNS) [8,10,11,12].

In obese animals, leptin plays an important role during SI; it stimulates the increase in inflammatory signaling following the immune challenge with LPS [13]. As a response to SI, the deep body temperature (Tb) is altered promoting the fever or hypothermia, depending on LPS dose. These changes in Tb occur in septic patients, as well as in rodents treated with LPS, even though we are currently far from fully understanding the mechanisms involved [14,15].

To minimize the deleterious effects of SI, therapeutic agents such as nonsteroidal anti-inflammatory drugs (NSAIDs) are commonly used in current therapy; however, the clinical use of NSAIDs is limited due to the gastrointestinal toxicity induced by these compounds [16,17,18]. Therefore, there is a great challenge of finding new therapeutic options for NSAIDs with lesser adverse effects in patients.

As an alternative, natural products are described as a major source of biomolecules with therapeutic potential, especially those of plant origin [19,20]. Citral (3,7-dimethyl-2,6-octadienal) is a racemic mixture of monoterpene isomers (neral and geranial) present as majority compound in the essential oils of *Cymbopogon citratus* and *Zingiber officinale* [21,22]. Reportedly, the anti-inflammatory, anti-hyperalgesic, antitumor, and antibacterial actions of citral have been investigated [23,24,25,26,27]. Citral demonstrated hepatoprotective effects against damage caused by HFD-induced obesity in mice [28], and, more recently, we have revealed the antipyretic role of citral in euthermic rats during SI mediated through the reduction of peripheric and central inflammatory mediators [29]. However, despite these important findings demonstrating the activities of citral, the effect of this monoterpene in SI modulation induced by LPS in HFD-fed obese mice remains unknown. In our study, we aimed to investigate the impact of citral on Tb and inflammatory signaling in obese mice during SI. Furthermore, we evaluated the impact of citral on body homeostasis during an LPS-induced immunological challenge.

## 2. Materials and Methods

### 2.1. Animals

Male Swiss mice (25–30 g, 5–6 weeks) were obtained from the Center of Animal Research and Production (Botucatu, SP, Brazil). Initially, animals were caged under controlled temperature (22 ± 1 °C) and light–dark cycle (12 h/12 h), with water and food provided ad libitum. All protocols were conducted between 7:00 a.m. and 4:00 p.m. Experimental protocols were approved the by the Ethics Committee for the Use of Animal of Biosciences Institute/UNESP (number 1080/2018 CEUA-IBB), in accordance with the Brazilian legislation regulated by the National Council for the Control of Animal Experimentation (CONCEA) and ethical principles in animal research formulated by the Brazilian Society of Science in Laboratory Animals.

### 2.2. Diet

The animals were divided into two groups, according to the diet offered, i.e., standard diet (SD) or HFD, for 12 weeks. The SD was composed of 15% lipids as a calorie source (Presence, Paulinia, SP, Brazil). The HFD contained 60% lipids as a calorie source for mice, as demonstrated in Table 1. All the components were purchased from the Prag Soluções Biociências company, Jaú, SP, Brazil.

### 2.3. Drugs

Citral (Sigma, St. Louis, MO, USA) was administered at three different doses (25, 100, and 300 mg/kg). Ibuprofen (IBU) (100 mg/kg; Cruz Vermelha Pharmacy of Manipulation, Botucatu, SP, Brazil) was used as a positive control NSAID; 1% Tween 80 was used as a vehicle for citral, IBU, and as a negative control. Tween 80 (Vetec, Rio de Janeiro, RJ, Brazil) was diluted in a pyrogen-free saline solution (0.9% NaCl), to a concentration of 1% (*w*/*v*). All treatments were administered orally (p.o.), by gavage (10 mL/kg).

*Escherichia coli* LPS (cat. 0111: B4, Sigma-Aldrich, St. Louis, MO, USA) was used for SI induction by intraperitoneal (i.p.) administration. LPS was dissolved and diluted in saline (100 µg/mL). The resulting solution was administered (1 mL/kg). Pyrogen-free saline was administered as a negative control (1 mL/kg).

### 2.4. Surgeries

After 12 weeks of obesity induction with SD and HFD, the mice were anesthetized (ketamine 87.5 mg/kg and xylazine 12.5 mg/kg, i.p.), to implant the temperature datalogger (SubCue, Calgary, AB, Canada) into the abdominal cavity. After the surgical procedure, pentabiotic (24,000 UI/kg, intramuscular; Zoetis, Fort Dodge Animal Health Ltd., Campinas, SP, Brazil) and an analgesic (2.5 mg/kg, subcutaneous; Flunixin meglumine, Chemitec Agro-Veterinária Ltd.a, São Paulo, SP, Brazil) were administered. For 72 h, the animals were maintained in individual cages for recovery before SI induction.

### 2.5. Measurements of Tb

Mice were maintained in the experimental room during surgical recovery. Tb was recorded at 5 min intervals throughout the experimental period (1 h before and 6 h after LPS administration). For each experimental group, the average of the initial Tb (Tbi) values (30 min before experimental manipulation) were used to determine the Tb variation (∆Tb) at 390 min [29]. In addition, the area under the curve was used to calculate the thermal index between 60 and 120 min. Animals fed SD and HFD were fasted for 8 h and acclimatized in the thermoneutral zone (30 ± 1 °C) for 12 h before SI induction [30]. The mice were subdivided into 5 groups: vehicle-1% Tween 80 (10 mL/kg-negative control), IBU (100 mg/kg-positive control NSAID), or citral (25, 100, and 300 mg/kg). The saline solution (1 mL/kg) or LPS (100 µg/kg) was injected 30 min after treatment. The mice were sacrificed at two different times: the first at 360 min after LPS injection for analysis of the Tb profile, and the second at 90 min after LPS injection, evaluating of the inflammatory signaling. Tb recordings were performed in non-anesthetized animals that were allowed free movement inside their respective cages.

### 2.6. Sample Collection for Morphometric and Molecular Parameters

After obtaining the Tb recordings, the animals were killed by decapitation, to collect the blood, brain, adipose tissues (retroperitoneal, visceral, and epididymal), liver, spleen, kidney, and heart. The adiposity index was determined by dividing the sum of adipose tissue weights by the final body weight. The brains were frozen by immersion in isopentane, cooled on dry ice, and stored at −80 °C. Blood samples were collected in serum-separator tubes and centrifuged at 3000× *g* for 15 min, at 25 °C. Serum samples (90 and 360 min after LPS injection) were then aliquoted and stored at −80 °C, until assay performance.

### 2.7. Hypothalamus Sample Collection

Using a matrix, the brains were positioned with the corpus mamillare facing upward. Hypothalamic samples were collected from two 500 μm thick slices, with samples from the lateral borders of the third ventricle, above the optic chiasm, and below the corpus callosum. The samples were homogenized in phosphate-buffered saline (PBS) 0.01M, pH 7.4, and 1% Protease Inhibitor Cocktail (PIC; Sigma, St. Louis, MO, USA) at a ratio of 1:9 (sample: PBS + PIC).

### 2.8. Measurement of Serum and Hypothalamic Levels of IL-1β, IL-6, and TNF-α

Serum and hypothalamus levels of cytokines (IL-1β, IL-6, and TNF-α) were determined by using the Multiplex Luminex Assay (Milliplex Multiplex Assays Luminex xMAP, Merck, Darmstadt, Germany) with Multiplex kits (cat. LXSAMSM-03, Magnetic Luminex Assay, mouse premixed Multi-Analyte Kit, R&D Systems, Minneapolis, MN, USA), following the manufacturer’s instructions.

### 2.9. Measurement of Serum Leptin Levels

The serum levels of leptin were determined by using an ELISA kit (cat. E-EL-M0039, Elabscience Biotechnology Inc., Houston, TX, USA), following the manufacturer’s instructions.

### 2.10. Measurement of Serum Biochemical Parameter Levels

Biochemical analyses were performed by using mouse serum post-euthanasia. The serum levels of glucose, urea, creatinine, aspartate aminotransferase (AST), alanine aminotransferase (ALT), gamma-glutamyltransferase (GGT), and alkaline phosphatase were determined, to evaluate glucose metabolism and kidney and liver damage, using colorimetric kits (BioClin, Belo Horizonte, MG, Brazil), for dry chemistry analysis, following the manufacturer’s instructions.

### 2.11. Evaluation of Inflammation and Oxidative Stress Parameters in Gastric Tissue

#### 2.11.1. Determination of Myeloperoxidase (MPO) Activity

MPO activity in the gastric mucosa was determined by using the method described by Krawisz and collaborators [31]. Tissue samples were solubilized in hexadecyltrimethylammonium bromide (HTAB) at a ratio of 1:20 (*w*/*v*). Then, the tissue solution was centrifuged (6540× *g* for 10 min at 4 °C), and 50 µL of the supernatant from each sample was obtained. MPO activity was determined through the sample reaction with 150 µL of o-dianisidine dihydrochloride (0.526 mM). The absorbance was determined at 450 nm, at 37 °C, using a spectrophotometer (BioTek Instruments Inc., Winooski, VT, USA). All data were expressed in units of MPO (U)/g of tissue.

#### 2.11.2. Determination of Malondialdehyde (MDA) Levels

The samples were homogenized in 1.15% KCl at a ratio of 1:5. The homogenate was centrifuged (7690× *g* for 10 min at 4 °C). Each test tube contained 1500 µL of 20% acetic acid (pH 3.5), 1500 µL of 0.8% 2-thiobarbituric acid (TBA) diluted in 20% acetic acid, 400 µL of distilled water, 200 µL of 8.1% SDS, and 400 µL supernatants of samples. After incubation in a water bath for 1 h, at 95 °C, the aliquot was centrifuged (1520× *g* for 10 min). The concentration of substances reacting with TBA was measured at 532 nm, and the results were expressed in µmol of MDA/g tissue [32].

#### 2.11.3. Determination of Reduced Glutathione (GSH) Levels

To quantify GSH levels, the method described by Anderson was used [33]. The GSH levels in the gastric samples were determined through homogenization in 5% trichloroacetic acid (TCA) buffer, at a ratio of 1:20, and centrifuged (1520× *g* for 10 min at 4 °C). Then, 20 µL of the supernatant was used to react with 140 µL of NADPH (0.1 mM), 20 µL of 5, 5′-dithiobis (2-nitrobenzoic acid) (DTNB, 10 mM), and 5 µL of PBS. The samples were incubated for 5 min, at 30 °C. Next, glutathione reductase (15 µL) was added to each sample. The absorbance was measured at 420 nm, and all data were expressed in nmol of GSH/g of tissue.

### 2.12. Statistical Analysis

Data are expressed as mean ± standard error of the mean (SEM). For comparison between two groups, the Student’s *t*-test was performed. For comparison between three or more groups, a one-way ANOVA followed by Dunnett’s or Tukey’s test, or two-way ANOVA, followed by Bonferroni’s test, were performed. The minimal significance level was set at a value of *p* < 0.05.

## 3. Results

### 3.1. Characterization of Morphometric Profiles in Eutrophic and Obese Animals after 12 Weeks

In this study, we used Swiss mice as a model to mimic obesity in humans. The initial mice body weights demonstrated no significant difference between groups, before SD or HFD feeding (*p* > 0.05). After 12 weeks, the HFD-fed animals presented a significant increase in body weight when compared with the SD group (Table 2). Additionally, our results demonstrated a significant increase in the adiposity index of mice fed with HFD when compared with the index observed in SD mice (Table 2). HFD-fed mice had a significant increase in the gross weight of liver, spleen, kidney, and adipose tissues (epididymal, retroperitoneal, and visceral) when compared with the SD-fed animals. Furthermore, we observed that, in HFD mice, the increased gross organ weights were accompanied by an increase in relative weight of adipose tissues and decrease in relative weight of kidney and heart when compared with SD mice (*p* < 0.05).

### 3.2. Tb Profile of Citral and Ibuprofen during 360 min after LPS-Induced SI in Eutrophic and Obese Mice

After 12 weeks of SD and HFD feeding to experimental mice, the effects of citral (25, 100, and 300 mg/kg) on LPS-induced SI were evaluated. Tb was assessed in all experimental groups for 360 min after LPS injection. Before LPS administration, no significant difference in Tbi was observed between groups (Table 3).

**Figure 1:**
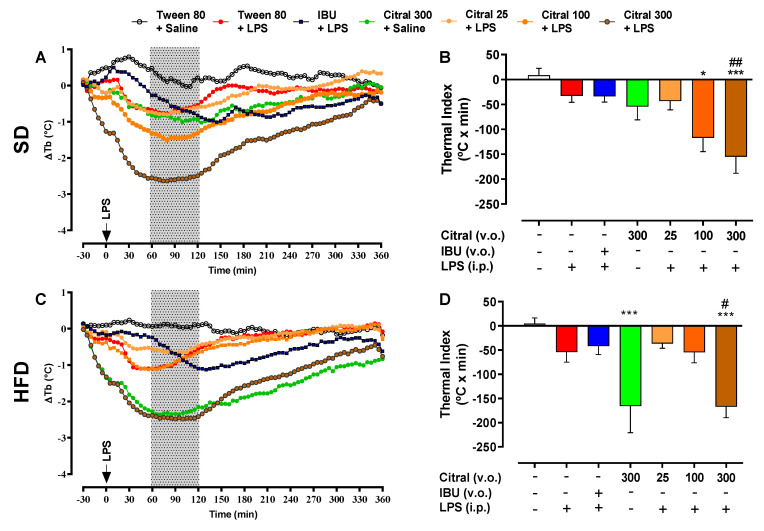
Effect of citral (25, 100, and 300 mg/kg) and IBU (100 mg/kg) oral treatment (p.o.) on body temperature variation (∆Tb) profile of mice fed standard diet (SD) and high-fat diet (HFD) after LPS-induced Systemic Inflammation (SI). Tb profile of eutrophic (**A**) and obese (**C**) mice was observed for 360 min after intraperitoneal (i.p.) administration of LPS (100 µg/kg) or saline (1 mL/kg). The hatched area represents the period used for calculating the thermal index of SD (**B**) and HFD (**D**) during 60–120 min of SI. The results are presented as mean ± SEM for *n* = 7–9 per group. One-way ANOVA followed by Tukey’s test; * *p* < 0.05 and *** *p* < 0.001 vs. Tween 80 + saline; # *p* < 0.05 and ## *p* < 0.01 vs. Tween 80 + LPS.

The injection of 100 µg/kg of LPS caused changes in Tb in all eutrophic (SD) and obese (HFD) mice (Figure 1). This variation was more representative between 60 and 120 min after LPS administration (gray area highlighted in Figure 1A,C). Eutrophic mice that received citral 300 + LPS (−154.90 ± 33.42 °C × min) demonstrated a significant reduction in ∆Tb when compared with the Tween 80 + LPS group (−32.70 ± 13.10 °C × min; Figure 1B). In contrast, both groups of obese mice treated with citral 300 + saline (−165.60 ± 55.07 °C × min) and citral 300 + LPS (−167.10 ± 22.44 °C × min) demonstrated significantly reduced thermal indices when compared with their respective control groups, Tween 80 + saline (4.66 ± 11.74 °C × min) and Tween 80 + LPS (−53.93 ± 20.96 °C × min), as demonstrated in the Figure 1D. Obese mice treated with the citral 300 + saline group showed a greater decrease in the thermal index during the period of 60 to 120 min after LPS administration when compared to eutrophic mice with the same treatment (Table 4).

### 3.3. Effect of Citral on Systemic Inflammatory Mediators after 90 min of LPS-Induced SI

In eutrophic and obese mice, the Tb reduction induced by citral after LPS injection was accompanied by changes in inflammatory parameters. In all experimental groups, inflammatory signaling during SI was assessed by quantifying serum levels of IL-1β, IL-6, TNF-α, and leptin (Table 5). After 90 min of LPS-induced SI, SD mice showed a significant increase in the serum levels of IL-6 and TNF-α when compared with the control group (Tween 80 + saline group). Additionally, the HFD mice of the Tween 80 + LPS group presented a significant increase in the serum levels of IL-1β, IL-6, and TNF-α when compared with the Tween 80 + saline group. Citral treatment (300 mg/kg) reduced the serum levels of TNF-α in SD mice 90 min after LPS administration when compared with the Tween 80 + LPS group. Additionally, in HFD mice, the same citral dose induced a significant reduction in serum levels of TNF-α and leptin after LPS administration when compared with the control group (Tween 80 + LPS group). In obese mice, the lowest dose of citral (25 mg/kg) reduced the serum levels of leptin, 90 min after LPS administration. In general, we observed that the HFD mice showed a significant increase in the serum levels of inflammatory mediators 90 min after LPS-induced SI when compared with the SD mice.

### 3.4. Effect of Citral on Hypothalamic Levels of Proinflammatory Cytokines after LPS-Induced SI

**Figure 2:**
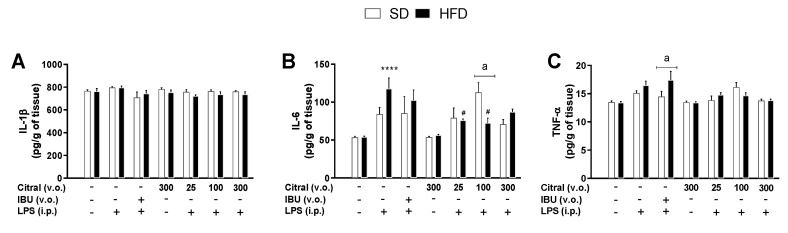
Effect of citral on the hypothalamic cytokine levels in eutrophic (SD) and obese (HFD) mice during systemic inflammation (SI). Hypothalamic levels of IL-1β (**A**), IL-6 (**B**), and TNF-α (**C**) after 90 min of LPS injection. The results are presented as mean ± SEM for *n* = 5–7 per group. One-way ANOVA followed by Tukey’s test; **** *p* < 0.001 vs. Tween 80 + saline; # *p* < 0.05 vs. Tween 80 + LPS. Two-way ANOVA followed by Bonferroni test; ^a^ = *p* < 0.05, compared to the SD mice.

During SI, the effect of citral on the hypothalamic levels of IL-1β, IL-6, and TNF-α were investigated 90 min after LPS injection (Figure 2). In SD mice, no changes were observed in hypothalamic cytokine levels in any experimental group. However, in obese mice, a significant increase was observed in the hypothalamic levels of IL-6 in the Tween 80 + LPS group (118.00 ± 13.90 pg/g of tissue) when compared with Tween 80 + saline (54.09 ± 1.06 pg/g of tissue; Figure 2B). Citral treatment caused a significant decrease in the hypothalamic IL-6 levels in obese mice administered citral 25 + LPS (75.98 ± 1.92 pg/g of tissue) and citral 100 + LPS (72.45 ± 6.55 pg/g of tissue) when compared with the Tween 80 + LPS group (Figure 2B). Additionally, our results demonstrated that the different diets interfered with the action of citral during hypothalamic inflammatory signaling in SI. After LPS administration, citral induced a reduction in hypothalamic IL-6 levels in obese mice when compared with eutrophic mice (SD = 113.10 ± 12.58 vs. HFD = 72.45 ± 6.55 pg/g of tissue; Figure 2B). Furthermore, treatment with IBU+LPS demonstrated a significant increase in hypothalamic TNF-α levels in obese mice when compared with eutrophic mice (SD = 14.47 ± 0.95 vs. HFD = 17.43 ± 1.52 pg/g of tissue; Figure 2C).

### 3.5. Biochemical Parameters during SI in Eutrophic and Obese Mice

In obese mice, citral treatment caused a significant increase in serum levels of glucose in the citral 100 + LPS group when compared with the same treatment in eutrophic mice. Moreover, citral treatment demonstrated no changes in urea and creatinine levels, indicating the absence of kidney damage after 360 min of LPS-induced SI in eutrophic and obese mice (Table 6).

Additionally, we investigated the effect of citral on serum levels of hepatic injury parameters after treatment and LPS-induced SI (Table 7). Citral treatment did not alter AST and GGT levels. In obese mice, citral treatment induced a significant increase in ALT levels in the citral 100 + LPS when compared with the control group (Tween 80 + saline). Furthermore, a significant decrease was observed in the alkaline phosphatase levels in both obese and eutrophic mice, independent of treatment.

### 3.6. Impact of Citral Treatment on the Gastric Mucosa of Eutrophic and Obese Mice

**Figure 3:**
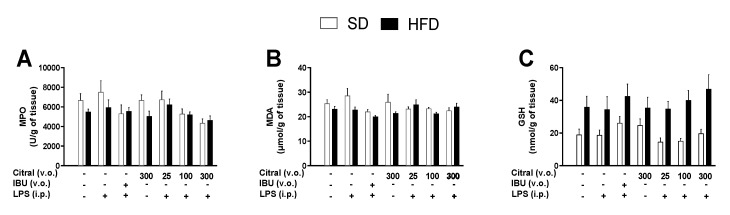
Effects of citral on local inflammation and oxidative stress on the gastric mucosa of animals fed standard diet (SD) and high-fat diet (HFD) after 360 min of the LPS-injection. We determined the MPO activity (**A**), as well as MDA (**B**) and GSH (**C**) levels, in eutrophic and obese mice. The results are presented as mean ± SEM for *n* = 7–9 per group. One-way ANOVA followed by Dunnett’s test. Two-way ANOVA followed by Bonferroni’s test.

Oral treatment of citral induced no alteration in the gastric mucosa after 360 min of LPS administration. In all experimental groups, the absence of changes in the gastric mucosa was assessed by macroscopic analysis after euthanasia (data not shown). This study evaluated additional parameters indicative of local inflammation and oxidative stress in the gastric mucosa of eutrophic and obese mice (Figure 3). Treatment with citral or IBU did not change MPO activity (Figure 3A), and MDA and GSH levels remained unaltered in the gastric tissue (Figure 3B and C, respectively,) when compared with the control group and diets.

## 4. Discussion

Obesity is a chronic disease caused by excessive food ingestion, provoking an increase in adipose tissue and metabolic disruption. The excessive accumulation of body fat contributes to the development of a low-grade chronic inflammatory status and plays a crucial role in the development of metabolic disorders associated with obesity [34]. In experimental animals, a model used to reproduce obesity involves offering a diet high in lipid content as the energy source [35]. This was first demonstrated by Samuels and collaborators, who induced obesity in rats with a diet containing 70% lipids, a condition similar to high caloric intake in humans [36]. In our study, we used a heterogeneous mouse strain and offered a HFD to induce obesity, mimicking the clinical conditions associated with this disease that impacts humans globally [37]. We observed that animals fed the HFD for 12 weeks showed an increase in body weight by approximately 36% when compared with SD-fed mice. This weight gain observed in HFD-fed animals, in addition to an increased adiposity index, revealed that obesity can be reproduced by eating saturated lipids inducing an increase in body fat accumulation [38,39,40]. The literature reveals that both parameters are in agreement with other studies realized with this same heterogeneous mouse strain, although some variations are present in body weight, duration, and diet composition [41]. The adiposity index considers body weight and the sum of adipose tissue and therefore presents fat accumulation in the body as an important indicator of effective obesity induction [42]. Hence, in the present study, the significant increase in these parameters in HFD mice, when compared with SD mice, confirms the establishment of the obesity model.

In obesity, a change in the inflammatory profile in all organisms occurs. The modulation of systemic proinflammatory pathways increases the production and release of inflammatory mediators, such as TNF-α and IL-6, mainly by the white adipose tissue [43]. The increase in systemic biomarkers promotes a meta-inflammation state with activation of immune system cells throughout the organism [44]. In obesity, low-grade chronic inflammation induces greater desensitization during an immunological response to pathogen infections [2,45]. Additionally, the literature described that obese individuals show an altered immune response and major infection susceptibility [45,46]. LPS-induced SI reproduces the responses observed during pathogen infection, promoting immunologic cell activation and subsequent production of peripheral and central inflammatory mediators [8]. Obesity promotes an increase in systemic levels of LPS (metabolic endotoxemia) and aggravates the SI response [47,48]. During SI, the production of inflammatory mediators causes Tb change, such as fever or hypothermia [15,49,50]. In the present study, we elucidated the thermoregulatory responses during SI after LPS injection in eutrophic and obese mice. Previous studies have described that the LPS-induced thermoregulatory response is modulated by two variables: the dose of the inductor agent and ambient temperature [30,51,52]. Firstly, the dose of the inductor agent can promote distinct responses, such as fever at low doses (0.5–100 µg/kg), and hypothermia, followed by fever at high doses (1–10 mg/kg) of LPS [30]. Moreover, during SI, the composition of the inductor agent, such as LPS, is directly associated with its effects [53]. Another factor related to the thermoregulatory response is the ambient temperature. This factor is responsible for preventing the activation of the metabolic mechanisms of Tb regulation (thermoneutral zone). The thermoneutral zone for mice is 30 °C [30]. In our study, all variables of the model were considered, with the administration of low-dose LPS and acclimatization of mice in the thermoneutral zone. However, we observed no change in Tb in the Tween 80 + LPS group when compared with the control group (Tween 80 + saline), in both dietary groups (eutrophic and obese mice). Previous investigations have demonstrated a significant reduction in Tb at 90 min after an immune challenge with LPS, when compared with animals receiving saline [29]. Furthermore, some studies have also reported that mice can show different thermoregulatory responses to low-dose LPS injection, such as the absence of fever [54]; additionally, hypothermia has also been described as a response to the immunological challenge with LPS [54,55], as was observed in our results.

Hypothermia is an acute effect of LPS injection, causing a significant increase in systemic inflammatory mediators. Endotoxin induces the inflammatory process through TLR-4 pathway activation in the organism periphery [55]. During LPS-induced SI, our results demonstrated a significant increase in serum TNF-α and IL-6 levels in both eutrophic and obese mice, corroborating with the validation of systemic inflammation induced by LPS in our model. Indeed, this inflammatory profile is related to the sensitization of macrophages and mast cells during SI induction by LPS [49]. TNF-α is a cytokine that plays an important role in immune–brain signaling [15]. This effect is particularly important in thermoregulatory responses during fever and hypothermia [56]. During the hypothermia response, this cytokine promotes a cryogenic effect when occurs inhibition of thermogenesis mechanisms without the activation of heat-loss mechanisms [57,58]. In obese animals, we observed a significant increase in serum levels of IL-6 and leptin after LPS injection when compared with eutrophic animals. Our results show that obese mice also present an increase in IL-1β levels when compared with eutrophic mice. This effect can be correlated with chronic low-grade inflammation during obesity because high levels of IL-1β (pyrogenic cytokine) in obese animals are directly associated with an adaptive subpyrogenic response of SI [34,59]. Moreover, some studies have demonstrated that the increase of IL-1β concentration, together with TNF-α, acts on signaling in the CNS regions (such as hypothalamic areas), to stimulate the synthesis of PGE_2_ during SI [49,60]. Other studies have shown the correlation between the levels of TNF-α and IL-1β mediating the release of leptin as an inflammatory response during SI [58]. In the CNS, we investigated the cytokine levels in the hypothalamus, the most important brain region responsible for thermoregulation. Our results demonstrated a significant increase in hypothalamic IL-6 concentrations in obese mice treated with Tween 80 + LPS when compared with the Tween 80 + saline group. This hypothalamic inflammatory signaling is typical in obese individuals fed a diet rich in lipids, which induces an increase in IL-6 and NF-κB gene expression, consequently causing neuronal death during obesity [61]. Currently, the best treatment used to minimize the effects of SI is NSAIDs, such as IBU. However, at high doses or when administered chronically, these drugs can cause adverse effects, such as epigastric pain and peptic ulcers [62]. Therefore, it is necessary to search safer and effective alternative drugs for the Tb variation caused by SI in different organism profiles, especially in obese patients in whom these drugs often have no effect [45].

In the present study, we demonstrated the hypothermic action of citral during SI in eutrophic and obese mice. We elucidated a new effect of high dose citral during LPS-induced SI, promoting a reduction in Tb and the thermal index in eutrophic and obese mice. Moreover, the hypothermic effect of citral occurs regardless of whether or not LPS is administered [63]. Our results showed that citral caused a significant reduction in Tb independent of LPS administration in obese mice when compared with eutrophic mice. We also investigated the inflammatory signaling profile after citral treatment as a mean to understand its action on LPS-induced SI modulation. Citral decreased systemic inflammatory mediators’ concentration, such as TNF-α levels in eutrophic mice, as well as TNF-α and leptin levels in obese mice. Citral probably acted during the initial SI phase, the period in which the serum TNF-α levels start to increase, signaling hypothalamic regions during SI [49,60]. In our previous studies, we have documented that citral reduces IL-1β, IL-6, TNF-α, and PGE_2_ plasma levels during LPS-induced fever in rats [29]. This immunomodulatory property of citral has already been described in the literature, and this effect occurs by the downregulation of inflammatory mediator signaling during local inflammatory responses in carrageenan-induced paw edema and a significant decrease in leukocyte recruitment in the peritoneal cavity of mice [64]. Gonçalves and collaborators [65] have recently reported the anti-inflammatory and antihyperalgesic activities of citral through the modulation of TLR-4, TLR-2/dectin-1 ligands, and cannabinoid receptor 2 (CB2R) in mice models. In these studies, the effect of citral on the modulation of inflammatory responses has been highlighted. However, no study has demonstrated the effect of citral on the reduction of peripheral mediators during LPS-induced SI in obese mice.

To the best of our knowledge, this is the first study to reveal the action of citral in reducing serum leptin levels during LPS-induced SI in obese mice. Leptin, a hormone with a conserved structure in correlation with other molecules, demonstrates signaling functions in the entire organism [66,67]. Particularly, leptin is important for homeostasis modulation and immunological functions. The literature described the action of leptin-mediated signaling associated with low food intake and reduced mobility during LPS-induced SI [47,68]. Pohl and collaborators [7] have reported that LPS administration causes an increase in circulating levels of the leptin in obese rats. All of these data demonstrated the crucial role of leptin in modulating the SI response in obese individuals. In our study, we demonstrated the important action of citral in promoting a reduction in serum leptin levels in obese mice. Some studies have reported that, during SI, elevated leptin levels have been observed in obese rats, along with increased production of IL-6 by astrocytes in the CNS [13]. This study suggests that, during SI, there is a direct relationship between leptin levels in the periphery and increased IL-6 levels in the CNS. We observed that acute citral administration caused a reduction in the hypothalamic IL-6 levels in obese mice, when compared with the control group, possibly associated with hypothermic mechanisms during SI. Citral is probably capable of crossing the blood–brain barrier, possibly due to the hydrophobic nature of the molecule and thus induce a neuroprotective effect during SI in obesity [65,69]. This property of citral could explain its ability to lower serum leptin levels and decrease hypothalamic IL-6 levels during SI. Therefore, these effects can be attributed to the hypothermic action of citral in obese mice, which has not been previously described. Furthermore, citral showed distinct actuation when compared with IBU, which only acts as a nonselective reversible inhibitor of cyclooxygenase 1 (COX-1) and COX-2 isoenzymes during SI [62]. Our results demonstrated that the administration of IBU induces a significant increase in the hypothalamus levels of TNF-α in obese mice when compared with eutrophic mice. This IBU response can be attributed to the need for dose increment in obese individuals necessary for its pharmacological action to occur, as observed at its clinical use [70]. To develop citral as an important pharmacological alternative for SI, it is necessary to better elucidate the signaling pathways in different individuals. This monoterpene may possess important clinical applications, possibly associated with nanoparticles that would improve its effectiveness as a drug with effects in hypothermia and neuroprotection [69].

In comparison to fever relatively little is known about the mechanisms responsible for hypothermia, even though SI-associated hypothermia is currently considered to have substantial clinical significance [71]. Hypothermia was considered a failure of the thermoregulatory effectors during severe SI. More recently, this notion has been reconsidered by studies documenting that specific thermoregulatory mechanisms take place in SI to induce hypothermia [72]. In the present study, we observed that citral enhanced hypothermia. Since hypothermia is now accepted as an adaptive response when the costs of fever exceed its benefits [72], we believe that citral has a potential to be therapeutically used as a drug to induced regulated hypothermia.

Regardless of these beneficial pharmacological attributes of citral treatment during SI, as demonstrated in our study, it remains crucial to evaluate the safety in eutrophic and obese animals [73]. Acute treatment with citral caused hyperglycemia in obese mice, and this effect is possibly associated with increased glucose mobilization during SI, which provides the energy for an inflammatory response [74]. Elevated serum glucose levels are an adaptive response to increased lipolysis, as well as protein breakdown induced by hepatic production and release of counter-regulatory hormones [74,75,76]. In clinical conditions, long periods of exposure to high glucose levels are associated with infection and sepsis, which can cause multiple organ failure and even death [74]. Citral caused no alteration in parameters associated with renal injury but changed the ALT serum levels. Other studies have documented the nontoxic effect of citral, such as Uchida and collaborators [77], which elucidated its effect in preventing acetaminophen-induced acute toxicity with the ability to reduce the serum levels of hepatic enzymes (AST, ALT, alkaline phosphatase, and GGT), decrease oxidative stress, and demonstrate an anti-inflammatory role in the hepatic tissue.

Furthermore, we investigated the effect of acute citral treatment on the gastric mucosa of animals after 360 min of LPS administration. We demonstrated that treatment with citral did not alter the integrity of the gastric mucosa after oral administration. However, the use of NSAIDs can induce ulcers and hemorrhages and, consequently, cause perforations in the gastrointestinal tract [78]. Thus, the parameters associated with local inflammation and oxidative stress were evaluated to better comprehend the effect of citral treatment on the gastric tissue. Our findings demonstrated that citral caused no alteration in MPO activities, an important indicator of neutrophil infiltration during local inflammation. Additionally, our results showed no alteration in MDA and GSH levels, indicative of lipidic peroxidation caused by oxidative stress and antioxidant mechanisms in the gastric mucosa, respectively [79]. However, it is necessary to highlight that the administration of anti-inflammatory drugs is not performed as a single dose [80]. Therefore, further studies with repeated citral doses are necessary to reproduce the effects in humans.

## 5. Conclusions

In summary, citral had a marked cryogenic effect during LPS-induced SI in eutrophic and obese mice. This effect of citral can be attributed to the reduction peripheral and central inflammatory mediators. Especially in obese mice, citral treatment reduced serum levels of TNF-α and leptin, which further promoted a reduction in hypothalamic IL-6 levels during SI. Acute citral treatment did not induce lesions in the gastric mucosa and did not alter the toxicity parameters, although the intermediate dose caused the alteration of a hepatic parameter. Thus, we demonstrated, for the first time, the effects of citral in an experimental model that reproduces SI in obese individuals, providing a possible therapeutic alternative for the treatment of obesity-associated diseases, such as infections and inflammatory processes.

## Figures and Tables

**Table 1:** Composition of the standard diet (SD) and high-fat diet (HFD).

Compounds	Standard Diet (SD)		High-Fat Diet (HFD)	
	g/kg	kcal/kg	g/kg	kcal/kg
Corn Starch	397.5	1590	115.5	462
Casein	200	800	200	800
Sucrose	100	400	100	400
Maltodextrin	132	528	132	528
Lard	-	-	312	2808
Soybean Oil	70	630	40	360
Cellulose	50	-	50	-
Mineral Mix	35	-	35	-
Vitamin Mix	10	-	10	-
L-Cysteine	3	-	3	-
Choline	2.5	-	2.5	-
Total	1000	3948	1000	5358

**Table 2:** Morphometric analysis of male Swiss mice fed with standard diet (SD) or high-fat diet (HFD) after 12 weeks.

	SD (*n =* 58)		HFD (*n =* 72)	
Initial body weight	30.00 ± 0.38 g		29.93 ± 0.26 g	
Final body weight	40.49 ± 0.48 g		51.04 ± 0.52 g ^a^	
Adiposity index	0.42 ± 0.03%		0.72 ± 0.02% ^a^	
	**Gross Weight (g)**	**Relative Weight (%)**	**Gross Weight (g)**	**Relative Weight (%)**
Liver	1.70 ± 0.03	4.18 ± 0.06	2.19 ± 0.06 ^a^	4.28 ± 0.10
Spleen	0.12 ± 0.00	0.30 ± 0.01	0.14 ± 0.01 ^a^	0.28 ± 0.01
Kidney	0.33 ± 0.01	0.79 ± 0.02	0.29 ± 0.01 ^a^	0.55 ± 0.01 ^a^
Heart	0.22 ± 0.00	0.56 ± 0.01	0.20 ± 0.00	0.39 ± 0.01 ^a^
Epididymal Adip. Tis.	0.85 ± 0.07	2.06 ± 0.15	1.40 ± 0.04 ^a^	2.75 ± 0.10 ^a^
Retroperitoneal Adip. Tis.	0.32 ± 0.03	0.76 ± 0.06	0.96 ± 0.05 ^a^	1.89 ± 0.09 ^a^
Visceral Adip. Tis.	0.53 ± 0.04	1.30 ± 0.08	1.32 ± 0.05 ^a^	2.57 ± 0.09 ^a^

All data are expressed as mean ± standard error of the mean (SEM). Student’s *t*-test; ^a^ = *p* < 0.05 compared to the SD mice.

**Table 3:** Initial body temperature (Tbi) of animals fed with standard diet (SD) or high-fat diet (HFD) 60 min before the LPS-induced Systemic Inflammation (SI).

Initial Body Temperature (Tbi, °C)							
	Tween 80 + Saline	Tween 80 + LPS	IBU + LPS	Citral 300 + Saline	Citral 25 + LPS	Citral 100 + LPS	Citral 300 + LPS
**SD**	35.66 ± 0.25	36.06 ± 0.30	35.84 ± 0.56	36.06 ± 0.22	35.98 ± 0.15	36.58 ± 0.29	36.26 ± 0.26
**HFD**	35.73 ± 0.23	36.36 ± 0.31	36.01 ± 0.25	36.08 ± 0.25	36.21 ± 0.26	36.42 ± 0.31	36.56 ± 0.16

All data are expressed as mean ± SEM for *n* = 7–9 per group. One-way analysis of variance (ANOVA) followed by Tukey’s test.

**Table 4:** Diet effect on the thermal index of animals with different treatments during LPS-induced Systemic Inflammation (SI).

Thermal Index (°C × min)		
Groups	SD	HFD
Tween 80 + Saline	8.65 ± 14.03	4.66 ± 11.74
Tween 80 + LPS	−32.70 ± 13.10	−53.93 ± 20.96
IBU 100 + LPS	−33.42 ± 11.80	−41.44 ± 17.94
Citral 300 + Saline	−54.31 ± 26.75	−165.60 ± 55.07 ^a^
Citral 25 + LPS	−43.21 ± 17.59	−36.40 ± 9.84
Citral 100 + LPS	−117.00 ± 27.45	−54.70 ± 21.68
Citral 300 + LPS	−154.90 ± 33.42	−167.10 ± 22.44

All data are expressed as mean ± SEM for *n* = 7–9 per group. Two-way ANOVA followed by Bonferroni’s test; ^a^ = *p* < 0.05, compared to the SD mice.

**Table 5:** Citral’s effect on the serum levels of cytokines (IL-1β, IL-6 and TNF-α) and leptin in animals fed with standard diet (SD) or high-fat diet (HFD) after 90 min of the LPS-injection.

**Groups**	**IL-1β (pg/mL)**		**IL-6 (pg/mL)**		**TNF-α (pg/mL)**		**Leptin (pg/mL)**	
	**SD**	**HFD**	**SD**	**HFD**	**SD**	**HFD**	**SD**	**HFD**
Tween 80 + Saline	8.14 ± 0.16	7.64 ± 0.01	68.21 ± 4.71	139.00 ± 15.33	1.08 ± 0.06	1.08 ± 0.01	0.33 ± 0.13	1.02 ± 0.19
Tween 80 + LPS	8.89 ± 0.16	9.61 ± 0.20 ****	54,468.00 ± 4401.00 ****	71,621.00 ± 8588.00 ^a^ ****	322.20 ± 72.29 ****	407.00 ± 34.51 ***	0.91 ± 0.23	1.74 ± 0.19 ^a^
IBU 100 + LPS	8.98 ± 0.20	10.12 ± 0.19 ^a^	50,232.00 ± 5352.00	86,452.00 ± 6368.00	415.70 ± 59.61	693.10 ± 92.64 ^a #^	0.92 ± 0.33	1.49 ± 0.26
Citral 300 + Saline	7.64 ± 0.00	8.44 ± 0.21	213.00 ± 60.83	161.30 ± 29.31	1.10 ± 0.07	1.40 ± 0.07	0.89 ± 0.32	0.79 ± 0.17
Citral 25 + LPS	9.03 ± 0.20	10.22 ± 0.22 ^a^	61,831.00 ± 5047.00	71,133.00 ± 9311.00	248.90 ± 35.80	462.00 ± 83.82 ^a^	0.82 ± 0.14	0.96 ± 0.10 ^#^
Citral 100 + LPS	9.57 ± 0.26	9.98 ± 0.39	80,850.00 ± 5015.00	50,871.00 ± 11,452.00	185.70 ± 23.92	221.90 ± 53.59	0.69 ± 0.06	0.88 ± 0.10
Citral 300 + LPS	9.09 ± 0.17	10.08 ± 0.31 ^a^	62,521.00 ± 8848.00	69,538.00 ± 11,459.00	99.84 ± 9.23 ^##^	139.40 ± 18.85 ^#^	0.62 ± 0.05	0.65 ± 0.10 ^##^

All data are expressed as mean ± SEM for *n* = 6–7 per group. One-way ANOVA, followed by Tukey test; * *p* < 0.05, ** *p* < 0.01, *** *p* < 0.001 and **** *p* < 0.0001 vs. Tween 80 + saline; # *p* < 0.05 and ## < 0.01 vs. Tween 80 + LPS. Two-way ANOVA followed by Bonferroni test; ^a^ = *p* < 0.05 compared to the SD mice.

**Table 6:** Effect of citral on biochemical parameters. Evaluation of the metabolic (glucose levels) and renal (urea and creatinine levels) function of eutrophic (SD) and obese (HFD) mice.

Groups	Glucose (mg/dL)		Urea (mg/dL)		Creatinine (mg/dL)	
	SD	HFD	SD	HFD	SD	HFD
Tween 80 + Saline	75.43 ± 14.21	88.60 ± 7.38	97.89 ± 31.38	82.23 ± 37.82	0.48 ± 0.14	0.49 ± 0.15
Tween 80 + LPS	69.43 ± 5.371	98.00 ± 18.91	72.33 ± 27.59	38.21 ± 5.45	0.37 ± 0.03	0.32 ± 0.02
IBU 100 + LPS	77.50 ± 6.68	84.14 ± 4.91	92.58 ± 38.95	69.79 ± 12.38	0.34 ± 0.05	0.45 ± 0.06
Citral 300 + Saline	89.86 ± 10.84	100.8 ± 15.94	89.91 ± 32.08	35.80 ± 2.34	0.43 ± 0.11	0.34 ± 0.01
Citral 25 + LPS	65.67 ± 3.31	75.14 ± 4.29	55.22 ± 23.61	40.19 ± 5.09	0.35 ± 0.05	0.34 ± 0.02
Citral 100 + LPS	65.33 ± 10.51	86.71 ± 6.18 ^a^	42.90 ± 8.70	48.36 ± 8.33	0.33 ± 0.02	0.32 ± 0.01
Citral 300 + LPS	80.86 ± 12.11	77.20 ± 4.72	105.90 ± 32.29	60.53 ± 16.78	0.50 ± 0.15	0.34 ± 0.03

All data are expressed as mean ± SEM for *n* = 6–8 per group. One-way ANOVA followed by Dunnett’s test. Two-way ANOVA followed by Bonferroni’s test; ^a^ = *p* < 0.05, compared to the SD mice.

**Table 7:** Effect of citral on biochemical parameters of hepatic function in eutrophic (SD) and obese mice (HFD).

Groups	AST (UI/L)		ALT (UI/L)		Alkaline Phosphatase (UI/L)		GGT (UI/L)	
	SD	HFD	SD	HFD	SD	HFD	SD	HFD
Tween 80 + Saline	113.60 ± 42.60	<2.0	63.29 ± 9.31	57.17 ± 4.01	37.57 ± 5.21	12.75 ± 0.48 a	<1.0	<1.0
Tween 80 + LPS	110.70 ± 39.61	<2.0	51.71 ± 14.91	96.14 ± 16.21	17.00 ± 3.01 *	11.57 ± 9.64	<1.0	<1.0
IBU 100 + LPS	<2.0	<2.0	72.83 ± 11.73	124.6 ± 29.08	21.40 ± 5.79	23.00 ± 0.01	<1.0	<1.0
Citral 300 + Saline	107.40 ± 44.79	<2.0	54.29 ± 11.26	92.25 ± 16.23	21.00 ± 1.86	10.67 ± 1.02	<1.0	<1.0
Citral 25 + LPS	111.20 ± 59.58	<2.0	81.83 ± 18.31	74.40 ± 6.56	22.00 ± 2.77	13.86 ± 0.86	<1.0	<1.0
Citral 100 + LPS	133.30 ± 61.01	<2.0	85.17 ± 26.22	227.30 ± 60.96 *	16.33 ± 2.09 *	17.43 ± 3.12	<1.0	<1.0
Citral 300 + LPS	114.10 ± 49.78	<2.0	59.71 ± 4.59	95.86 ± 20.26	26.14 ± 3.73	13.67 ± 2.22	<1.0	<1.0

All data are expressed as mean ± SEM for *n* = 6–8 per group. One-way ANOVA followed by Dunnett’s test; * *p* < 0.05 vs. Tween 80 + saline. Two-way ANOVA followed by Bonferroni’s test; ^a^ = *p* < 0.05 compared to the SD mice.
